# A Hebbian cell assembly based neural field model for the remote associate task and creative search

**DOI:** 10.1186/1471-2202-16-S1-P284

**Published:** 2015-12-18

**Authors:** Ivana Kajic, Thomas Wennekers

**Affiliations:** 1 School of Computing and Mathematics, Plymouth University, Plymouth, Devon PL48AA, UK

## 

The Remote Associates Test (RAT) is widely used in experimental psychology to assess verbal creative thinking [1]. Subjects are given triplets of words (the 'cues', e.g., 'spoon', 'coin', and 'quick') and instructed to find a fourth word that relates to all the cues (the 'target', e.g., 'silver'). Cues presented separately usually elicit strong associations with numerous other words (close associates), but since their combination triggers the specific target word, it is said that this target is 'remotely' associated. Several studies have addressed the cognitive search process in RAT, which, given sufficient search time, can go through a number of candidate words before the target is actually found (e.g., [2]).

In order to elucidate possible neural mechanisms underlying the RAT, we here propose a neural field model that combines the idea of Hebbian cell assemblies (CAs) with a winner-take-all competitive process for assembly selection (WTA), and inhibition-of-return (IOR) [3] to allow for multiply-constrained autonomous searches on word-networks. Words are represented by localised populations of neurons (assemblies) mapped over the 2D model sheet; they can be activated by inputs representing the cues. Semantic similarity is implemented by the lateral network of hetero-associative connections in such a way that strongly related words have stronger mutual, possibly asymmetric, connections. Local excitatory connections support the amplification and latching of activity in an assembly. Local inhibition, evoked by activity in an assembly, can overcome the excitation of that assembly after some time, switch it off, and provide a lasting inhibition-of-return. This allows for the sampling of other potential target words. Global inhibition (WTA) furthermore keeps the total activity in the network small, such that at any time only a small number of CAs can be active.

We present computer simulations of the search process in a space of associated words under different conditions regarding association strength between close and remote associates, search time limits, and the impact of noise on RAT performance. We also analyse the network dynamics to determine conditions that allow for the existence of remote associates and that support a Markovian search dynamic as described experimentally [2].
